# *N*′-Substituted 4-Phenylpicolinohydrazonamides with Thiosemicarbazone Moiety as New Potential Antitubercular Agents: Synthesis, Structure and Evaluation of Antimicrobial Activity

**DOI:** 10.3390/ma15165513

**Published:** 2022-08-11

**Authors:** Katarzyna Gobis, Małgorzata Szczesio, Andrzej Olczak, Ida Mazerant-Politowicz, Dagmara Ziembicka, Barbara Pacholczyk-Sienicka, Ewa Augustynowicz-Kopeć, Agnieszka Głogowska, Izabela Korona-Głowniak, Andrzej Fruziński

**Affiliations:** 1 Department of Organic Chemistry, Faculty of Pharmacy, Medical University of Gdańsk, 107 Gen. Hallera Ave., 80-416 Gdańsk, Poland; 2 Institute of General and Ecological Chemistry, Faculty of Chemistry, Lodz University of Technology, Żeromskiego 116, 90-924 Lodz, Poland; 3 Institute of Organic Chemistry, Lodz University of Technology, Żeromskiego 116, 90-924 Lodz, Poland; 4 Department of Microbiology, Institute of Tuberculosis and Pulmonary Diseases, 26 Płocka Str., 01-138 Warsaw, Poland; 5 Department of Pharmaceutical Microbiology, Faculty of Pharmacy, Medical University of Lublin, 1 Chodźki Street, 20-093 Lublin, Poland

**Keywords:** picolinohydrazonamide, thiosemicarbazones, synthesis, temperature NMR, X-ray, ADME, tuberculostatic activity, antimicrobial activity

## Abstract

Three new 4-phenylpicolin derivatives with a thiosemicarbazone structure were synthesized and evaluated for tuberculostatic activity. The compounds were obtained by the condensation of methyl 4-phenylpicolonimidate with the corresponding cycloalkylamino-1-carbothiohydrazides. The ^1^H NMR temperature spectra obtained showed proton lability at the nitrogen atom N2, and X-ray crystallography confirmed the zwitterionic structure of all products. ADME calculations indicate that the compounds can be tested as future drugs. All compounds were absorbed in the gastrointestinal tract. All compounds also showed very good tuberculostatic activity (MIC 3.1–12.5 µg/mL). Derivative **1b** showed the best selectivity for *M. tuberculosis* compared to the other pathogenic species tested. The study has allowed the emergence of imine derivative **1b** as a good structure for further optimization in the search for antitubercular drugs.

## 1. Introduction

Tuberculosis is one of the leading causes of death worldwide. It is an infectious disease declared a global public health emergency in 1993 by the WHO. Until the COVID-19 outbreak, tuberculosis was the leading infectious killer, surpassing HIV/AIDS [1,2]. It is a treatable disease, but its treatment regimen is complicated, as one should always use a combination of at least two drugs over a long period of time. This is done in order to increase the effectiveness of treatment as well as prevention of formation of resistant forms [3]. Mycobacteria can mutate on their own and develop resistance to the drugs used [4,5]. This happens as a result of improper treatment, e.g., inadequate duration of therapy, incorrectly selected medications, incorrect dose, or incomplete treatment cycle. In order to prevent this unfavorable phenomenon, the rules of pharmacotherapy should be scrupulously followed [6,7]. Due to the fact that tuberculosis is still one of the diseases leading to death and is a threat mainly due to the constantly developing chemotherapeutic and antibiotic resistant strains of *Mycobacterium tuberculosis*, new, effective anti-tuberculosis agents are being sought [8,9], especially given that the most effective anti-tuberculosis drug is still isoniazid, which was introduced into medical treatment in 1952 [10].

**Figure 1:**
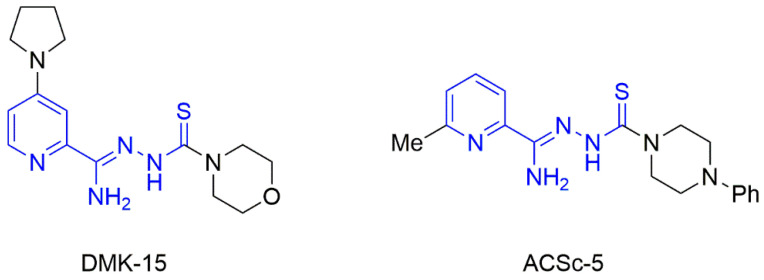
Structures of picolinohydrazonamide **DMK-15** and **ACSc-5**.

Previously, we described the synthesis and antimicrobial properties of the cycloalkylamine-1-thiosemicarbazone derivatives of picolinohydrazonamide **DMK-15** and **ACSc-5** (Figure 1) [11,12]. The first one, with two rings of secondary cyclic amines, pyrrolidine and morpholine, showed tuberculostatic activity towards *M. tuberculosis* at the MIC (minimal inhibition concentration) level of 0.4 µg/mL with low cytotoxicity against the human dermal fibroblast line HDF, with IC50 (half-maximal inhibitory concentration) of 36.18 µg/mL. The second one, with a methyl group in the C-6 position and a phenylpiperazine fragment at the end of the thiosemicarbazone substituent, showed considerable activity against the *Staphylococcus aureus* ATCC 25923 strain (MIC 15.6 µg/mL) and the *Bacillus subtilis* ATCC 6633 strain (MIC 7.8 µg/mL). These results show that the combination of a pyridine ring and a thiosemicarbazone moiety in one molecule can result in good antimicrobial activity without adversely affecting eukaryotic cells. Thus, further structural studies, both in solid and liquid states, seemed to be a crucial element in the search for the influence of tautomerism on the antimicrobial activity of thiosemicarbazone compounds.

In this research, we describe the synthesis of three new 4-phenylpicoline derivatives with thiosemicarbazone structure. The interesting analytical results obtained for these products prompted us to study their structure thoroughly. We carried out ^1^H NMR temperature studies and X-ray crystallography. The compounds showed remarkable activity against both standard and resistant strains when tested with *M. tuberculosis* species. Then they were tested for the activity towards other species of pathogenic microorganisms. Compounds were also analyzed for their pharmacokinetic properties, drug similarity, and absorption.

## 2. Materials and Methods

### 2.1. Chemistry

The reagents, solvents, and materials used were of analytical purity (Sigma-Aldrich-Merck KgaA, Darmstadt, Germany). The purity of the products was confirmed by thin-layer chromatography (TLC). In the analysis, silica gel plates were used, and a lamp emitting light with a wavelength of λ = 254 nm was used for detection. The melting points of the compounds obtained were measured once with a Stuart SMP30 (Stone, Staffordshire, UK). IR spectra for potassium bromide pellets of solids were recorded on a Satellite FT-IR spectrophotometer (Bruker, Madison, WI, USA). Standard ^1^H and ^13^C NMR spectra were performed in CDCl_3_ or DMSO-*d*_6_ using Varian Unity Plus (500 MHz) and Varian Gemini (200 MHz) instruments (Varian Medical Systems, Palo Alto, CA, USA). The ^1^H NMR spectra of **1a**–**c** were acquired at various temperatures (233, 243, 253, 263, 273 and 300 K) using a Bruker Avance II Plus 16.4 T spectrometer (Bruker BioSpin, Rheinstetten, Germany) operating at an ^1^H frequency (700.16 MHz). The elemental composition of the products was determined on the basis of elemental analysis (% C, H, N), and the obtained results were consistent with the values calculated with a maximum deviation of 0.4%.

Methyl 4-phenylpicolinimidate was synthesized according to method described by us elsewhere [13] and analytical data were consistent with those obtained by other authors [14].

#### 2.1.1. General Procedure for the Synthesis of Hydrazonamides (**1a**–**c**)

Methyl 4-phenylpicolinimidate (0.36 g, 2 mmol) was dissolved in methanol (5 mL) and treated with DBU (0.35 mL, 2.3 mmol). The solution was refluxed for 30 min and then the appropriate cycloalkylamino-1-carbothiohydrazide (2 mmol) was added without cooling the mixture. The mixture was refluxed another 15 min, cooled in an ice bath and the precipitated products were filtered off and recrystallized from ethanol, giving yellow crystals. In the case of compound **1c**, the ice was also added to the mixture.

##### 4-Phenyl-*N*′-(pyrrolidine-1-carbonothioyl) Picolinohydrazonamide (**1a**)

From pyrrolidine-1-carbothiohydrazide (0.29 g), the compound **1a** was obtained (0.23 g, 46%): m.p. 173–175 °C; IR: 3419, 3274 (υ N-H), 3149, 3031, 2950, 2865 (υ C-H), 1669, 1601 (υ C=N), 1463, 1430 (υ C=C), 1362, 1298, 1264, 1237 (υ C-N), 763, 696 (γ C-H) cm^−1^; ^1^H NMR (500 MHz, DMSO-*d*_6_): δ 1.85–1.86 (m, 4H, 2CH_2_), 3.56–3.60 (m, 4H, 2CH_2_), 6.85 (br. s, 2H, NH_2_ + D_2_O exchangeable), 7.55–7.61 (m, 4H, 3H Ph and 1H pyridine), 7.97–7.98 (m, 2H, Ph), 8.53 (s, 1H, pyridine), 8.81 (d, 1H, pyridine, J = 5 Hz), 12.62 (s, 1H, NH + D_2_O exchangeable) ppm; ^13^C NMR (175 MHz): δ 25.26 (2C), 48.61 (2C), 118.92, 123.66, 127.59 (2C), 129.76 (2C), 130.49, 136.60, 143.76, 145.31, 149.40, 150.89, 176.48 ppm; Anal. Calcd for C_17_H_19_N_5_S (325.14): C, 62.74; H, 5.88; N, 21.52; Found: C, 62.95; H, 6.15; N, 21.19.

##### *N*′-(Morpholine-4-carbonothioyl)-4-phenylpicolinohydrazonamide (**1b**)

From morpholine-4-carbothiohydrazide (0.32 g), the compound **1b** was obtained (0.55 g, 80%): m.p. 171–173 °C; IR: 3282 (υ N-H), 3155, 3044, 2953, 2922, 2836 (υ C-H), 1671 (υ C=N), 1589, 1463, 1414 (υ C=C), 1344, 1259, 1220 (υ C-N), 1116 (υ C-O), 1023 (δ C-H), 892, 767 (γ C-H) cm^−1^; ^1^H NMR (200 MHz, CDCl_3_): δ 3.74 (t, 4H, 2CH_2_, J = 5 Hz), 3.98 (t, 4H, 2CH_2_, J = 5Hz), 6.75 (br. s, 2H, NH_2_ + D_2_O exchangeable), 7.51–7.68 (m, 6H, 5H Ph and 1H pyridine), 8.11 (s, 1H, pyridine), 8.70 (d, 1H, pyridine, J = 5 Hz) 12.53 (s, 1H, NH) ppm; ^13^C NMR (175 MHz, DMSO-*d*_6_): δ 47.19 (2C), 66.72 (2C), 119.21, 123.98, 127.60 (2C), 129.78 (2C), 130.53, 136.51, 145.18, 145.24, 149.45, 150.94, 179.22 ppm; Anal. Calcd for C_17_H_19_N_5_OS (341.13): C, 59.80; H, 5.61; N, 20.51; Found: C, 59.44; H, 5.45; N, 20.15.

##### 4-Phenyl-*N*′-(4-phenylpiperazine-1-carbonothioyl) Picolinohydrazonamide (**1c**)

From 4-phenylpiperazine-1-carbothiohydrazide (0.47 g), the compound **1c** was obtained (0.37 g, 45%): m.p. 178–180 °C; IR: 3266 (υ N-H), 3149, 3024, 2843 (υ C-H), 1668, 1598 (υ C=N), 1495, 1463, 1413 (υ C=C), 1339, 1223 (υ C-N), 1018 (δ C-H), 763, 693 (γ C-H) cm^−1^; ^1^H NMR (500 MHz, DMSO-*d*_6_): δ 3.13–3.15 (m, 4H, 2CH_2_), 4.04 (m, 4H, 2CH_2_), 6.81 (t, 1H, Ph, J = 7 Hz), 7.00 (d, 2H, Ph, J = 8 Hz), 7.24 (t, 2H, Ph, J = 7 Hz), 7.54–7.60 (m, 3H, Ph), 7.87 (br. s, 1H, NH + D_2_O exchangeable), 7.96–7.98 (m, 3H, 2H Ph and 1H pyridine), 8.57 (s, 1H, pyridine), 8.76–9.26 (m, 2H, 1H pyridine and 1H NH + D_2_O exchangeable), 12.69 (s, 1H, NH + D_2_O exchangeable) ppm; ^13^C NMR (175 MHz): δ 46.34 (2C), 49.04 (2C), 116.23 (2C), 119.23, 119.50, 123.95, 127.60 (2C), 129.42 (2C), 129.77 (2C), 130.50, 136.56, 145.15, 145.27, 149.47, 150.93, 151.70, 179.10 ppm; Anal. Calcd for C_23_H_24_N_6_S (416.18): C, 66.32; H, 5.81; N, 20.18; Found: C, 66.28; H, 5.41; N, 19.86.

### 2.2. X-ray Study

X-ray diffraction experiments of the studied compounds were done on a diffractometer (Bruker SMART APEXII CCD) using CuKα radiation. Diffraction data were processed with SAINT ver. 8.34A, SADABS ver. 2014/4 (structure 1c with TWINABS ver. 2008/4) and XPREP ver. 2014/2. The structures were solved with the ShelXT (Version 2018/2) [15] and refined with ShelXL 2018/3 [16]. For visualization, ShelXle [17] and Olex2 [18] programs were used. Structure 1c was refined as a three-component twin with the following contributions: 0.88, 0.11, and 0.01. All H atoms (except those in NH and NH2 groups) were geometrically optimized. All studied crystals were obtained by slow evaporation of solvent. The solvent used is a mixture of methanol and DMF (1:1). Programs publCIF [19] and Mercury [20] were used to prepare the manuscript. CCDC 2189843, 2189844, 2189856 contain the supplementary crystallographic data for this paper. The data were provided free of charge by the Cambridge Crystallographic Data Centre via www.ccdc.cam.ac.uk/structures (accessed on 13 July 2022).

### 2.3. ADME

The compounds were analyzed for their pharmacokinetic properties, drug-likeness and absorption. An ADME analysis done with SwissADME service [21], a free web tool to evaluate pharmacokinetics, drug-likeness molecules and BOILED-Egg to predict gastrointestinal absorption and brain penetration of molecules [22].

### 2.4. Tuberculostatic Activity Assay

The newly synthesized hydrazonamides were tested in vitro for their anti-tuberculosis activity against three strains of *M. tuberculosis*: H37Rv and two native strains isolated from patients of the National Institute of Tuberculosis and Lung Diseases in Warsaw, Poland. Strain Spec. 210 was resistant to clinically used anti-tuberculosis drugs: *p*-aminosalicylic acid (PAS), isoniazid (INH), ethambutol (ETB), and rifampicin (RMP). Spec. 192 showed full sensitivity. The tests were carried out using the classic test tube method described in detail earlier [23]. For the compound **1b**, which showed the highest activity during the tests, the research was extended to include non-tuberculous strains *M. bovis*, *M. kanssasi*, *M. intracellulare*, and *M. scrafulaceum*. Each test was performed in triplicate. Representative data are presented.

### 2.5. In Vitro Antibacterial Activity Assay

Compounds **1a**–**c** were screened for antibacterial and antifungal activities by microdilution broth method as described elsewhere [24]. For all compounds, minimal inhibition concentration (MIC), minimal bactericidal concentration (MBC), and minimal fungicidal concentration (MFC) were determined. The tested derivatives were evaluated for the panel of the reference microorganisms from American Type Culture Collection (ATCC), including Gram-positive bacteria (*Staphylococcus aureus* ATCC 25923, *Staphylococcus aureus* ATCC 43300, *Staphylococcus aureus* ATCC 6538, *Staphylococcus epidermidis* ATCC 12228, *Micrococcus luteus* ATCC 10240, *Bacillus cereus* ATCC 10876, *Bacillus subtilis* ATCC 6633, *Streptococcus pyogenes* ATCC 19615, *Streptococcus mutans* ATCC 25175, *Streptococcus pneumoniae* ATCC 49619),Gram negative bacteria (*Salmonella* Typhimurium ATCC14028, *Escherichia coli* ATCC 25922, *Klebsiella pneumoniae* ATCC 13883, *Pseudomonas aeruginosa* ATCC 9027, *Proteus mirabilis* ATCC 12453), and fungi (*Candida parapsilosis* ATCC 22019, *Candida albicans* ATCC 10231, *Candida parapsilosis* ATCC 22019) (LGC Standards, Teddington, Middlesex, UK). Each experiment was performed in triplicate. Representative data are presented.

## 3. Results and Discussion

### 3.1. Chemistry

**Figure 2:**
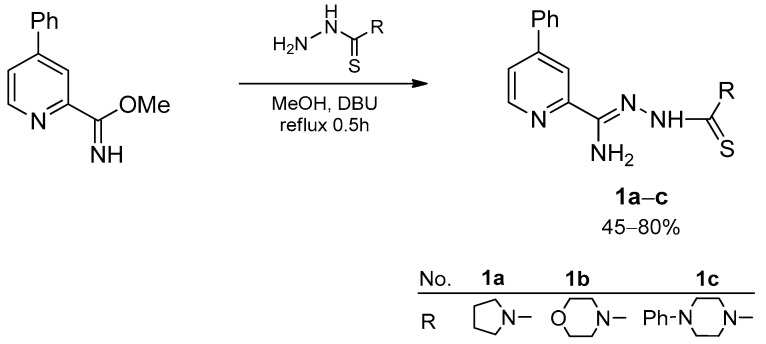
Synthesis of compounds **1a**–**c**.

The initial compound 4-phenyl-2-cyanopyridine refluxed for 1 h with 1,8-diazabicyclo[5.4.0]undec-7-ene (DBU) in methanol gave methyl 4-phenylpicolinimidate with 85% yield (Figure 2) [13]. The addition of DBU is intended to prevent the substitution at position 4 of the pyridine ring. Received compound was refluxed with cycloalkylamino-1-carbothiohydrazides for 15 min to the hydrazonamides **1a**–**c**. Cycloamino-1-carbothiohydrazides were obtained according to modified method described by Klayman et al. [25]. The substituents used were pyrrolidine, morpholine, and 4-phenylpiperazine, the reaction yield depended on the cyclic amine (45–80%).

**Figure 3:**
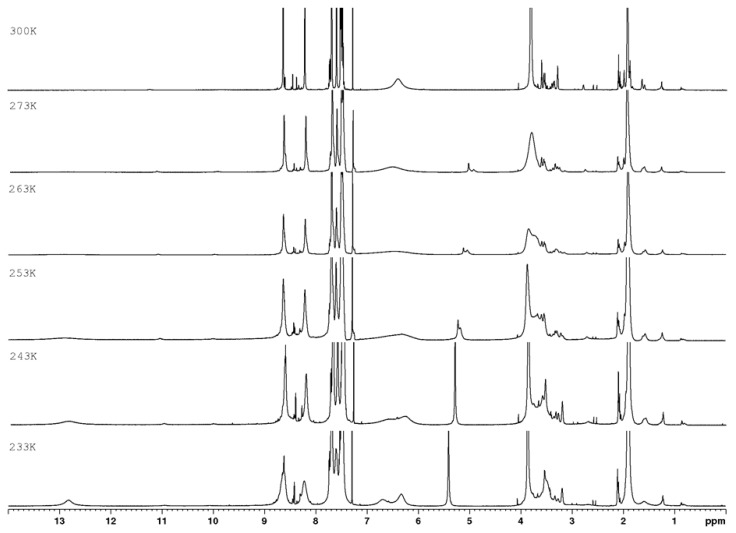
^1^H NMR spectra of **1a** as a function of temperature (700 MHz, CDCl_3_).

**Figure 4:**
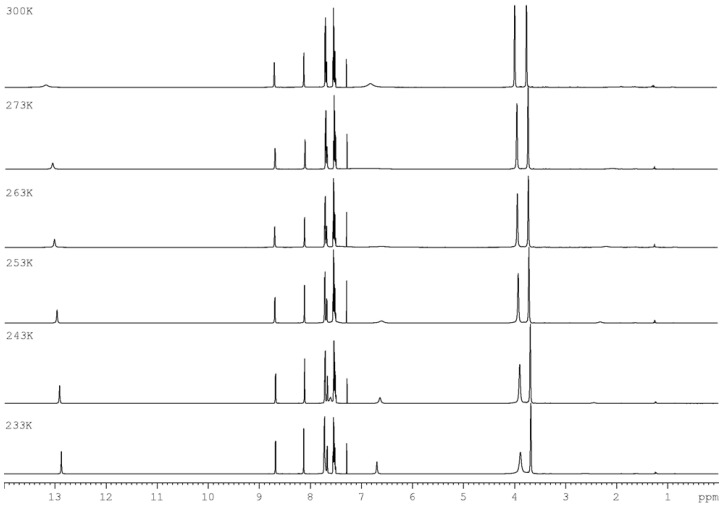
^1^H NMR spectra of **1b** as a function of temperature (700 MHz, CDCl_3_).

**Figure 5:**
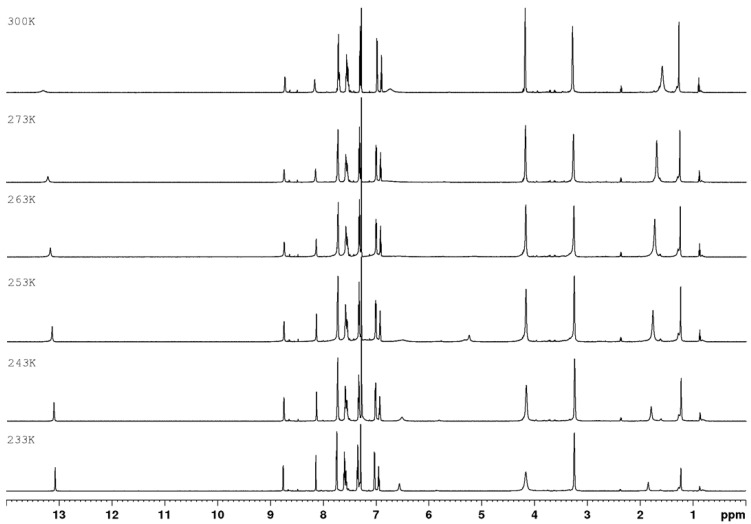
^1^H NMR spectra of **1c** as a function of temperature (700 MHz, CDCl_3_).

**Figure 6:**
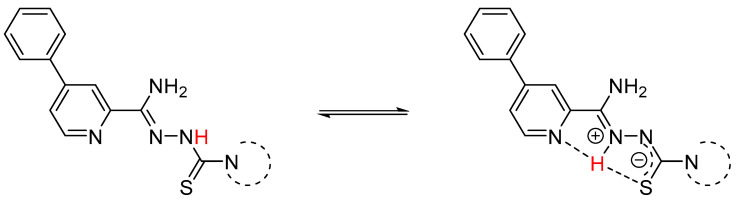
Tautomeric forms of compounds **1a**–**c**. The labile hydrogen atom is marked in red.

In ^1^H NMR experiments recorded for compounds **1a**–**c** at various temperatures (Figure 3, Figure 4 and Figure 5), it can be seen that the NH proton with a chemical shift of ~13.31 ppm at 300 K is very broadened and almost disappears in noise (Figure 6). As the temperature decreases, we get one sharp signal, which underwent an upfield shift (12.87 ppm). Changing the position of the labile proton at nitrogen atoms and the possibility of hydrogen bonding of this proton with the nitrogen atom in the aromatic ring changes the chemical environment of the NH_2_ protons. The consequence of this is the magnetic inequality of protons in the amino group and the appearance of separate signals on the spectrum at 243 K, while at 300 K there is one average signal (~6.40 ppm). Both signals undergo exchange upon heavy water. 

### 3.2. X-ray Study

**Figure 7:**
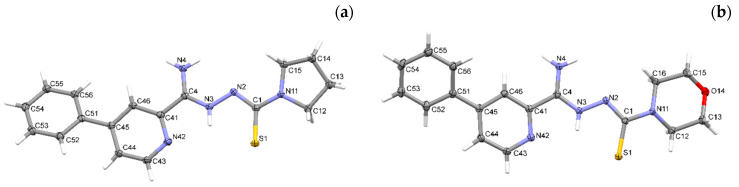

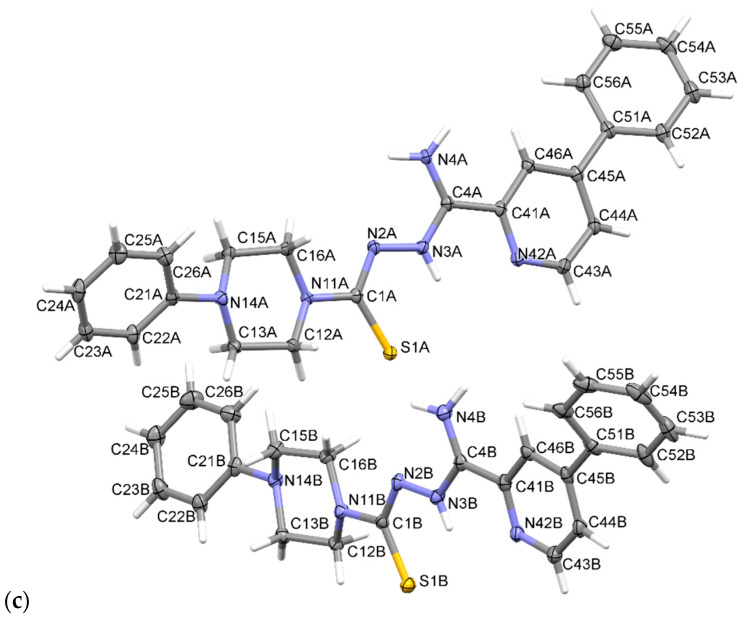
The molecular structures of compounds (**a**–**c**), with atom-labeling schemes. Displacement ellipsoids are drawn at the 50% probability level, and H atoms are shown as small spheres of arbitrary radii. Drawings were prepared with Mercury software.

**Figure 8:**
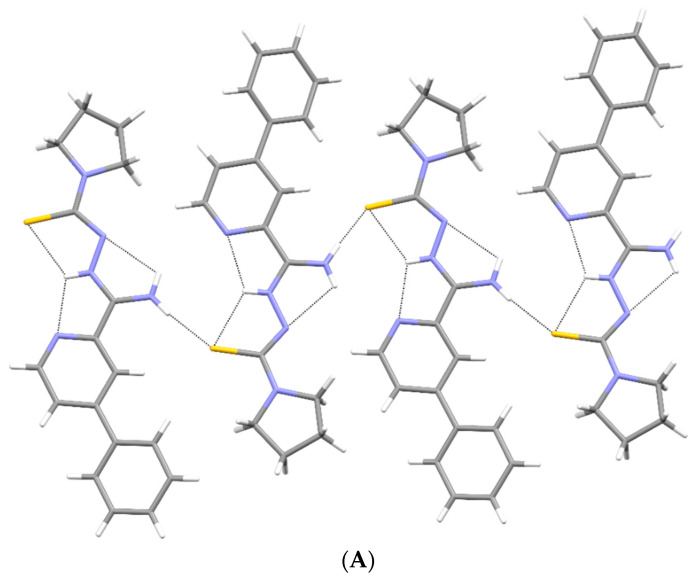

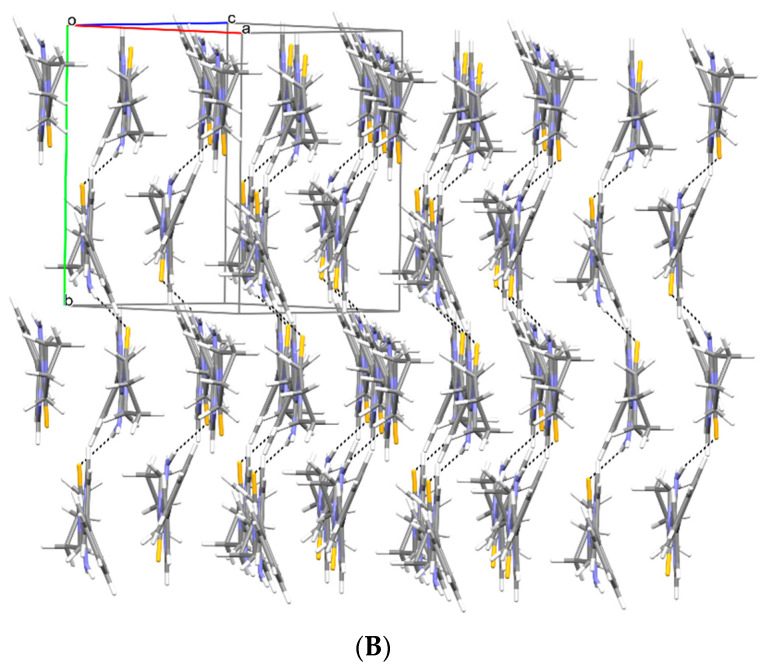
(**A**) Intermolecular hydrogen bonds in **1a**. (**B**) The packing of molecules in **1a** (a, b, c—unit cell).

**Figure 9:**
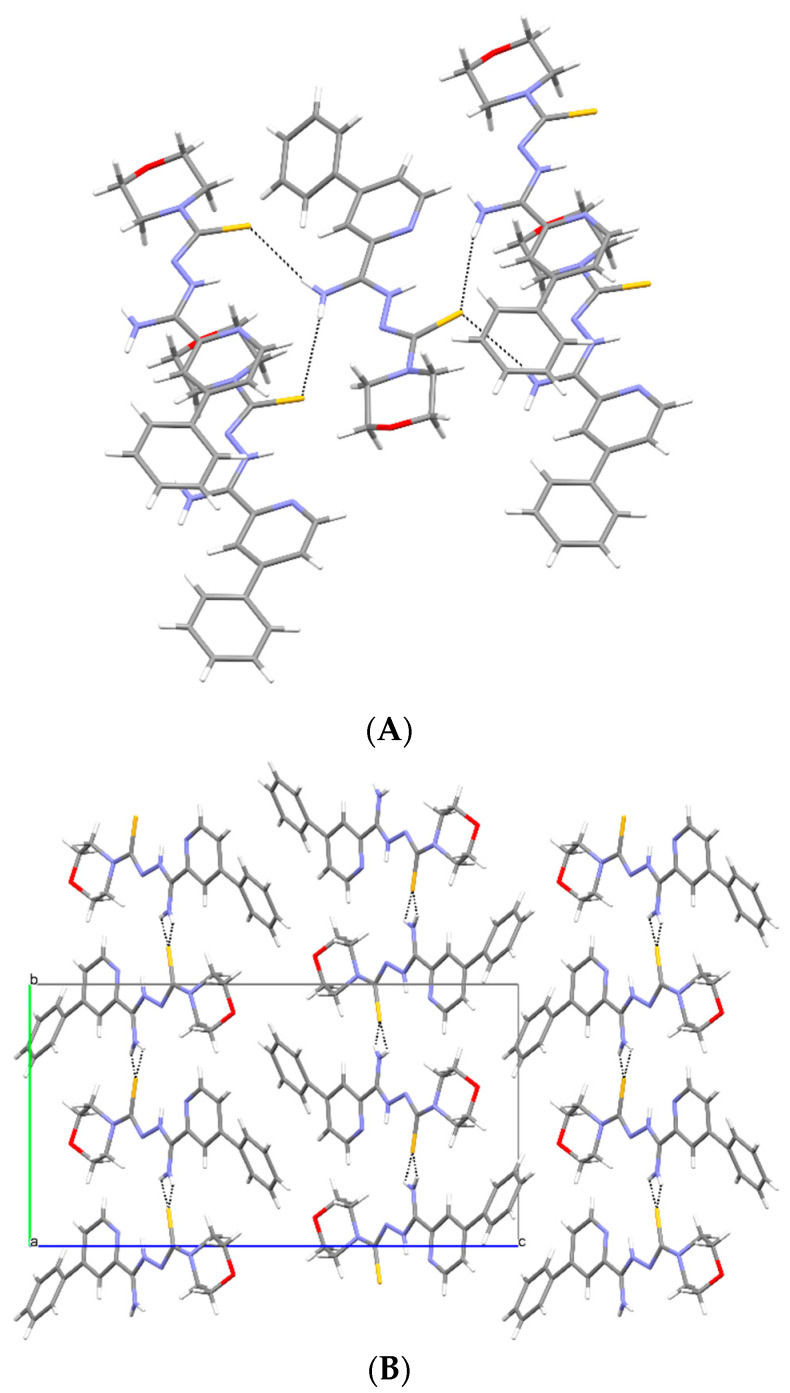
(**A**) Intermolecular hydrogen bonds in **1b**. (**B**) The packing of molecules in **1b** (a, b, c—unit cell).

**Figure 10:**
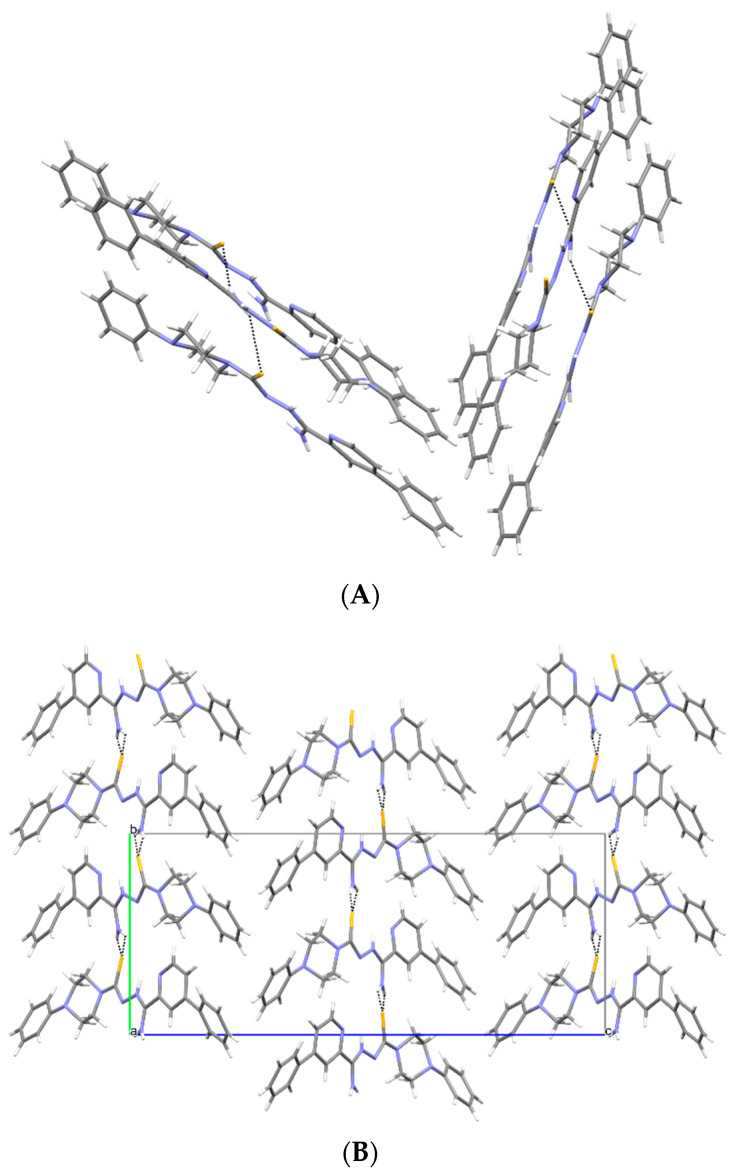
(**A**) Intermolecular hydrogen bonds in **1c**. (**B**) The packing of molecules in **1c** (a, b, c—unit cell).

Crystallographic data are presented in Table 1. All compounds crystallized in a monoclinic system. The compound **1c** has two molecules in the asymmetric unit and was refined as a three-component twin, and the other two compounds have one molecule in the asymmetric unit (Figure 7). All compounds take the form of zwitterion. The compound **1a** forms a chain of hydrogen bonds, and its conformation is stabilized by three intramolecular hydrogen bonds (Figure 8A). Infinite chains of hydrogen bonds strengthen the layered packing system (Figure 8B). Planar structure of molecules in compounds **1b** and **1c** are also intramolecular hydrogen bonds stabilizing the structure. In crystal structures of **1b** and **1c**, both hydrogen atoms of the NH_2_ group form hydrogen bonds of the N-H S type, creating a three-dimensional structure. Both compounds form a similar packing (Figure 9 and Figure 10).

**Figure 11:**
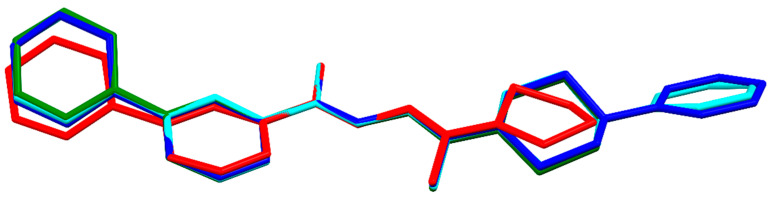
Superimposition of molecules for all determined structures; **1a**—red, **1b**—green, **1c**—blue/turquoise. H atoms were omitted for clarity.

The superposition of the molecules (Figure 11) indicates that the zwitterion form stabilizes the stretched form, and all compounds assume a similar conformation.

### 3.3. ADME

**Figure 12:**
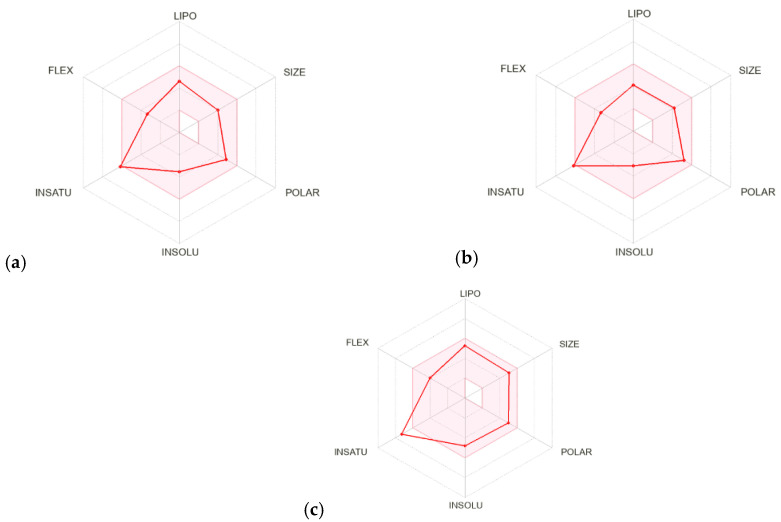
Bioavailability radar for (**a**–**c**).

**Figure 13:**
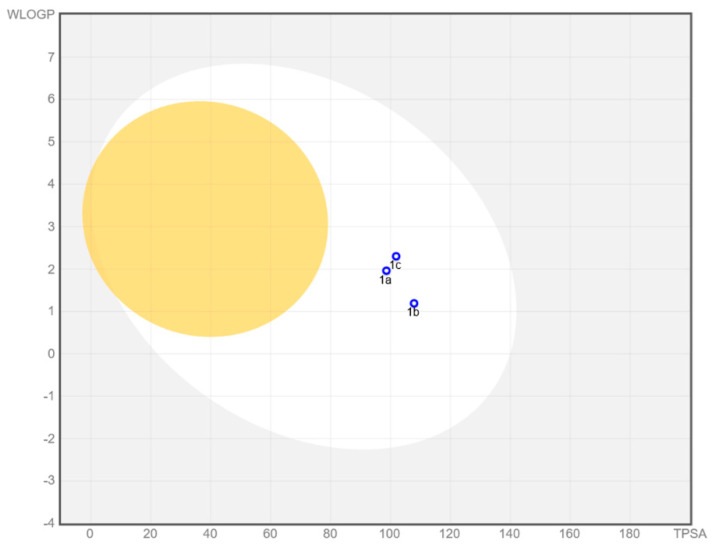
BOILED-Egg diagram for all compounds (lipophilicity (WLOGP) and polarity (tPSA), human intestinal absorption (white area) and blood–brain barrier permeation (yellow area).

Bioavailability radars for all studied compounds were made (Figure 12). For drug-like properties, the compounds were found to have a good bioavailability score (0.55). The compounds **1a** and **1b** meets with the rules of Lipinski [27], Ghose [28], Egan [29], Veber [30] and Muegge [31]. Compound **1c** does not fulfill Ghose’s rules. All compounds are good drug candidates. In the BOILED-Egg diagram (Figure 13), all compounds are absorbed in the gastrointestinal tract, which may make it an effective drug. No compounds permeate the blood–brain barrier.

### 3.4. Tuberculostatic Activity

The obtained compounds were tested for in vitro tuberculostatic activity against *M. tuberculosis*. The MIC values were defined as the minimum concentration inhibiting the growth of the tuberculosis strains tested relative to the control without the test compound. INH and PZA were used as reference drugs. The tested derivatives showed diversified activity (Table 2). However, two of the obtained compounds showed better activity than not only the reference PZA, which showed activity against the standard H37Rv strain and susceptible strain Spec. 192 at the level of MIC value 25 µg/mL and against the resistance strain Spec. 210 over 400 µg/mL, but also INH (MICs respectively 12.5 µg/mL and 25 µg/mL. Two derivatives (**1a** and **1b**) showed high activity toward all strain types (MIC 3.1–12.5 µg/mL). The presence of hydrophilic cyclic amine (pyrrolidine or morpholine) increased the activity, while the presence of highly lipophilic phenylpiperazine moiety (**1c**) significantly decreased the antitubercular potency (MIC 50 µg/mL). It is noteworthy that mostly the MIC values against the resistant strain were at the same level as against the standard strain.

The extended non-tuberculous assays have revealed that compound **1b** shows weaker inhibition of non-tuberculous mycobacteria (Table 3). MIC for *M. kansasii*, *M. intracellulare* and *M. scrofulaceum* was in the range of 6.2–12.5 µg/mL. The activity against *M. bovis* was merely at the same level as for tuberculous mycobacteria (3.1 µg/mL).

### 3.5. Antimicrobial Activity

Extensive microbiological tests were conducted for all the newly synthesized compounds (**1a**–**c**). The antibacterial and antifungal activities of tested compounds are presented in Table 4. Vancomycin (Van), ciprofloxacin (Cip) and nystatin (Nys) were used as the standard drugs. Markedly, the tested compounds showed mostly bacteriostatic activity (MBC/MIC ratio > 4). The minimal inhibition concentration (MIC) values for Gram-positive reference bacteria, indicated very strong (MIC < 10 mg/L) anti-staphylococcal (*S. aureus, S. epidermidis*), anti-micrococcal (*M. luteus*) and good (MIC 25–125 mg/L) anti-streptococcal *(S. pyogenes, S. pneumoniae, S. mutans*) activity of tested compounds [32]. Good bioactivity was also observed against Gram-negative bacteria (*S. typhimurium*, *E. coli*, *K. pneumoniae*, *P. mirabilis*, *P. aeruginosa,*) with MIC of 25–125 mg/L. Compound **1a** revealed the best antibacterial efficiency against Gram-positive bacteria. The most sensitive to tested compounds were staphylococcal strains. Antifungal bioactivity (*C. albicans, C. parapsilosis*) of tested compounds was determined as very strong (**1a**) and strong (**1b**, **1c**).

## 4. Conclusions

In conclusion, three new 4-phenylpyridine thiosemicarbazone derivatives were successfully synthesized from methyl 4-phenylpicolinimidate and cycloalkylamino-1-carbothiohydrazides. Spectroscopic analysis and structural crystallography allowed us to identify the labile proton at the nitrogen atom N2. In the crystalline state, these take the extended form of zwitterions. The planar conformation is stabilized by intramolecular hydrogen bonds. The packing of molecules in space is influenced by hydrogen bonds of the N-H S type. ADME calculations indicate that the compounds can be tested as future drugs. All compounds are absorbed in the gastrointestinal tract. This is a significant advantage, especially for potential anti-tuberculosis drugs, due to long-term therapy and patient comfort. Studies of tuberculostatic activity showed significantly high activity of the newly obtained derivatives. Extended tests for compound **1b** against non-tuberculous species of the *Mycobacterium* genus showed a broad spectrum of activity of this compound. At the same time, this compound showed the best selectivity of all the products obtained, having a lower effect on other species of pathogenic microorganisms. The most important finding, therefore, is that—due to its high tuberculostatic potential—compound **1b** may be a good starting structure for further research into new antimicrobial drugs.

## Figures and Tables

**Table 1:** Crystal data, data collection, and refinement details.

	1a	1b	1c
Crystal Data			
Chemical formula	C_17_H_19_N_5_S	C_17_H_19_N_5_OS	2(C_23_H_24_N_6_S)
*M*_r_	325.43	341.43	833.08
Space group	*P*2_1_/*c*	*P*2_1_/*c*	*P*2_1_
a, b, c (Å)	13.3591 (4), 12.0691 (4), 9.9817 (3)	5.9419 (4), 12.1487 (7), 22.9938 (13)	6.2510 (3), 11.9035 (5), 27.8679 (12)
β (°)	101.248 (2)	96.549 (2)	93.696 (2)
*V* (Å^3^)	1578.46 (9)	1649.01 (17)	2069.30 (16)
*Z*	4	4	2
μ (mm^−1^)	1.87	1.86	1.56
Crystal size (mm)	0.87 × 0.24 × 0.20	1.24 × 0.18 × 0.14	1.0 × 0.41 × 0.13
Data Collection			
No. of measured, independent and observed [*I* > 2σ(*I*)] reflections	21324, 3119, 2979	17762, 3213, 3185	8030, 8030, 8024
(sin θ/λ)_max_ (Å^−1^)	0.618	0.618	0.618
Refinement			
*R*[*F*^2^ > 2σ(*F*^2^)], *wR*(*F*^2^), *S*	0.028, 0.076, 1.05	0.027, 0.073, 1.04	0.026, 0.074, 1.13
No. of reflections	3119	3213	8030
No. of parameters	220	230	568
No. of restraints	0	0	1
Δ_max_, Δ_min_ (e Å^−3^)	0.22, −0.32	0.34, −0.18	0.20, −0.19
Absolute structure	–	–	Flack x determined using 3129 quotients [(I+) − (I-)]/[(I+) + (I-)] [26]
Absolute structure parameter	–	–	0.046 (4)

**Table 2:** In vitro tuberculostatic activity of compounds **1a–c** ^1,2,3^.

Compd.	MIC ^1^ [µg/mL]		
	H_37_Rv ^2^	Spec. 192	Spec. 210
**1a**	**6.2**	**12.5**	**6.2**
**1b**	**3.1**	**3.1**	**3.1**
**1c**	50	50	50
**INH ^3^**	12.5	12.5	25
**PZA ^3^**	25	25	>400

The values obtained for the most potent compounds are marked in bold. ^1^ Minimal inhibiting concentration has been determined by the classical test-tube method of successive dilution. ^2^ *M. tuberculosis* H_37_Rv, Spec. 192, Spec. 210. ^3^ **INH** isoniazid; **PZA** pyrazinamide.

**Table 3:** In vitro activity of compound **1b** towards various *Mycobacterium* strains.

*Mycobacterium* Strain	MIC [µg/mL]
*M. tuberculosis* H_37_Rv	3.1
*M. bovis*	3.1
*M. kanssasi*	12.5
*M. intracellulare*	6.2
*M. scrafulaceum*	12.5

**Table 4:** In vitro antimicrobial activity of compounds **1a**–**c** against reference bacterial and fungal strains ^1,2,3^.

	1a			1b			1c			
	MIC	MBC	MBC/MIC Ratio	MIC	MBC	MBC/MIC Ratio	MIC	MBC	MBC/MIC Ratio	MIC
Gram-Positive Bacteria										**Van**
*S. aureus* ATCC 25923	1.95	15.6	8	1.95	1.95	1	0.98	31.3	32	0.98
*S. aureus* ATCC 43300	3.9	125	32	1.95	250	128	0.49	31.3	64	0.49
*S. aureus* ATCC 6538	3.9	62.5	16	1.95	125	64	1.95	31.3	16	0.49
*S. epidermidis* ATCC 12228	1.95	125	64	0.98	31.3	16	0.49	31.3	64	0.98
*M. luteus* ATCC 10240	0.49	15.6	32	1.95	1.95	1	0.06	15.6	260	0.12
*B. cereus* ATCC 10876	3.9	31.3	8	7.8	>1000	>128	31.3	31.3	1	0.98
*B. subtilis* ATCC 6633	3.9	62.5	16	0.98	0.98	1	31.3	31.3	1	0.24
*S. pyogenes* ATCC 19615	7.8	15.6	2	15.6	31.3	2	62.5	125	2	0.24
*S. mutans* ATCC 25175	3.9	15.6	4	31.3	>1000	>32	62.5	62.5	1	0.98
*S. pneumoniae* ATCC 49619	7.8	31.3	4	31.3	31.3	1	31.3	125	4	0.24
Gram-Negative Bacteria										**Cip**
*E. coli* ATCC 25922	1000	>1000	Nd	125	>1000	>8	125	>1000	>8	0.015
*S. typhimurium* ATCC 14028	250	>1000	>4	125	>1000	>8	125	>1000	>8	0.061
*K. pneumoniae* ATCC 13883	>1000	>1000	Nd	500	>1000	Nd	125	>1000	>8	0.122
*P. mirabilis* ATCC 12453	125	>1000	>8	31.3	>1000	>32	62.5	>1000	>16	0.030
*P. aeruginosa* ATCC 9027	500	>1000	Nd	31.3	>1000	>32	62.5	>1000	>16	0.488
Yeasts	MIC	MFC		MIC	MFC		MIC	MFC		**Nys**
*C. albicans* ATCC 102231	15.6	62.5	4	15.6	62.5	2	7.8	7.8	1	0.48
*C. parapsilosis* ATCC 22019	62.5	62.5	1	15.6	125	8	7.8	31.3	4	0.24

^1^ MIC—minimal inhibitory concentration, MBC—minimal bactericidal concentration, MFC—minimal fungicidal concentration. ^2^ **CIP** ciprofloxacin, **VAN** vancomycin, **Nys** nystatin. ^3^ Nd not detected

## Data Availability

Data is contained within the article.

## Abbreviations

a, b, c (Å)	unit cell parameters in angstroms
ACSc-5	(Z)-6-methyl-*N*′-(4-phenylpiperazine-1-carbonothioyl) picolinohydrazonamide
ADME	absorption, distribution, metabolism, and excretion
AIDS	acquired immune deficiency syndrome
ATCC	American Type Culture Collection
CCDC	Cambridge Crystallographic Data Centre
Cip	ciprofloxacin
COVID-19	Coronavirus Disease 2019
DBU	1,8-diazabicyclo [5.4.0]undec-7-ene
DMK-15	(*Z*)-*N*′-(morpholine-4-carbonothioyl)-4-(pyrrolidin-1-yl)picolinohydrazonamide
DMSO-*d*_6_	isotopologue of dimethyl sulfoxide
ETB	ethambutol
F2	structure factor squared
FLEX	flexibility
FT-IR	Fourier-transform infrared spectroscopy
H_37_Rv	standard *M. tuberculosis* strain
HDF	human dermal fibroblast line
HIV	human immunodeficiency virus
IC50	half-maximal inhibitory concentration
INH	isoniazid
INSATU	insaturation
INSOLU	insolubility
IR	infrared radiation
*M. tuberculosis*	*Mycobacterium tuberculosis*
MBC	minimal bactericidal concentration
MFC	minimal fungicidal concentration
MIC	minimal inhibition concentration
*M*_r_	molecular mass
NMR	nuclear magnetic resonance
Nys	nystatin
PAS	*p*-aminosalicylic acid
POLAR	polarity
PZA	pyrazinamide
*R*	index based on structure factors describing compatibility of the model with X-ray diffraction data
RMP	rifampicin
*S*	goodness of fit of the refined X-ray structure
Spec. 192	strain fully sensitive to the administrated tuberculostatic drugs
Spec. 210	strain isolated from tuberculosis patients resistant to PAS, INH, ETB, and RMP
tPSA	polarity
UV	ultraviolet
*V* (Å^3^)	volume of a unit cell in cube angstroms
Van	vancomycin
WHO	World Health Organization
WLOGP	lipophilicity
